# Evaluation of the acceptability and usefulness of an information website for caregivers of people with bipolar disorder

**DOI:** 10.1186/1741-7015-11-162

**Published:** 2013-07-11

**Authors:** Lesley Berk, Michael Berk, Seetal Dodd, Claire Kelly, Stefan Cvetkovski, Anthony Francis Jorm

**Affiliations:** 1 IMPACT Strategic Research Centre, School of Medicine, Deakin University, Geelong, Victoria, Australia; 2 Orygen Youth Health Research Centre, Centre for Youth Mental Health, University of Melbourne, Parkville, Victoria, Australia; 3 Department of Psychiatry, University of Melbourne, Parkville, Victoria, Australia; 4 Swanston Centre, Barwon Health, Geelong, Victoria, Australia; 5 Florey Institute for Neuroscience and Mental Health, University of Melbourne, Parkville, Victoria, Australia; 6 Mental Health First Aid, Parkville, Victoria, Australia; 7 Melbourne School of Population Health, University of Melbourne, Parkville, Victoria, Australia; 8 School of Psychology, Deakin University, Melbourne, Victoria, Australia

**Keywords:** Bipolar disorder, Caregiver burden, Caregivers, Control appraisals, Disseminate guidelines, Evaluation by users, Guidelines for caregivers, Information website, Website evaluation, Website for caregivers

## Abstract

**Background:**

Bipolar disorder is associated with extreme mood symptoms, disability and suicide risk. Close family or friends often have a primary role in supporting an adult with bipolar disorder. However, not all support is helpful and there is little publicly accessible evidence-based information to guide caregivers. Caregiver burden increases the risk of caregiver depression and health problems. To help fill the information gap, expert clinicians, caregivers and consumers contributed to the development of guidelines for caregivers of adults with bipolar disorder using the Delphi consensus method. This paper reports on an evaluation of the acceptability and usefulness of the online version of the guidelines, http://www.bipolarcaregivers.org.

**Methods:**

Visitors to the website responded to an initial online survey about the usefulness of the information (N = 536). A more detailed follow-up feedback survey was emailed to web users who were adult caregivers of adults with bipolar disorder a month later (N = 121). The feedback was analyzed quantitatively and qualitatively to establish user appraisals of the online information, whether and how caregivers applied the information and ways it could be improved.

**Results:**

The majority of users (86.4% to 97.4%) found the various sections of the website useful. At follow-up, nearly 93% of caregivers reported that the information was relevant to them and 96% thought it would help others. Most respondents said that the information was supportive and encouraged adaptive control appraisals. However, a few respondents who were experiencing complex family problems, or who cared for a person with severe chronic bipolar disorder did not appraise it as positively. Nevertheless, over two-thirds of the caregivers reported using the information. Optional interactive features were recommended to maximize benefits.

**Conclusions:**

Overall, http://www.bipolarcaregivers.org was appraised positively and used. It appears useful to close family and friends seeking basic information and reassurance, and may be an inexpensive way to disseminate guidelines for caregivers. Those who care for people with more severe and chronic bipolar disorder, or who have complex family problems might benefit from more specialized interventions, suggesting the importance of a stepped-care approach to supporting caregivers. The potential of evidence-based, collaboratively developed information websites to enhance caregiver and consumer outcomes merits further investigation.

## Background

Consumers and caregivers have highlighted the value of having a reliable information resource to refer to over the course of a chronic illness [1,2]. With the emphasis on community care for people with mental health problems, close family and friends often have a primary responsibility for supporting an adult with bipolar disorder. These caregivers may face many challenges given that people with bipolar disorder commonly spend at least half their time actively symptomatic, particularly with disabling depressive symptoms, have a high suicide risk and can engage in risky manic behavior [3,4]. Negative social, occupational and financial illness consequences can affect both the patient and their caregiver [5,6]. However, caregivers report a lack of information about how to deal with the person’s illness, and the changes and losses that commonly ensue, and feel isolated, alone and unsupported [7,8] Health professionals do not always have time to provide information for caregivers and confidentiality concerns compound the difficulty of implementing collaborative family-friendly models [9-11]. Nevertheless, caregivers need accessible, relevant information to help them to support the person with bipolar disorder and deal with the effect the illness can have on their own life.

Caregiver burden is considered to involve the objective illness-related-demands and problems related to the caregiving situation as well as the caregiver’s subjective experience of distress and emotional strain [12,13]. Although some caregivers see a positive side to caring, most experience some degree of burden due to illness-related problem behaviors (89% to 91.9% of caregivers), the disruptive effects of the illness on their work, social and leisure activities (61% to 82% of caregivers) and the person’s role dysfunction (52% to 65%) [6,14,15]. Highly burdened caregivers tend to neglect their basic self-care and risk depression and physical health problems that increases their reliance on health services [16]. Many people with bipolar disorder have commented on the valuable role close family or friends can play in helping them to manage their illness and maintain a good quality of life, but caregivers need information about ways to do this without jeopardizing their own health [17,18].

The caregiver’s lack of knowledge about bipolar disorder can exacerbate negative consequences for both themselves and the person with bipolar disorder. For example, conflict and distress may result when caregivers misinterpret the person’s symptoms as part of the person’s deliberate difficult behavior (for example, viewing lethargy as laziness) [19,20]. Caregivers can feel very distressed when they recognize how ill the person is, but don’t know how to help or to control the situation [14]. They may resort to unhelpful avoidant coping and high expressed emotion [21,22]. In addition, if the caregiver is uninformed, they may discourage helpful illness management strategies such as the person’s medication adherence [23]. Poor support and high expressed emotion are linked to bipolar disorder relapse [24]. Thus, the caregiver’s lack of knowledge and burden can undermine their valuable supportive role.

Psychosocial interventions that have been evaluated in randomized controlled trials offer psychoeducation and training for family members or caregivers in ways to deal with bipolar disorder, but are seldom accessible to the public due to cost, expertise or resource limitations [25]. The quality of information on publicly available bipolar disorder or mental health websites is variable [26], and the content is more orientated to consumers than caregivers. Similarly, evidence-based clinical guidelines have been adapted for consumers and caregivers, but mainly provide information on bipolar disorder and its treatment rather than caregiving issues [27,28]. Thus, we conducted a Delphi consensus study, using the existing literature as a base and enlisting the views of panels of caregivers, clinicians and consumers with experience and expertise in dealing with bipolar disorder (n = 143) to develop guidelines for adult caregivers of adults with bipolar disorder. More detail about the development of the guidelines can be found in a previous article [29].

Guidelines need to be disseminated and used if they are to have any effect on health outcomes [30,31]. The internet is a convenient way of disseminating health information in developed countries where around 73.8% to 80% of people access the internet [32-34] and many caregivers search online for information about the health condition of a loved one [33,35]. Online information is accessed privately when convenient, quickly and easily, even in remote areas [36]. Thus, the guidelines developed in the Delphi study formed the content of the website http://www.bipolarcaregivers.org.

Although studies have highlighted the advantages of tailored interactive health information [37,38], for pragmatic reasons, we developed a simple static information website with text, pictures and graphics. In order to maximize user engagement, we consulted the literature about what consumers and caregivers themselves appreciate about the content, design and the way information is conveyed on health-related websites [39-41]. For example, the consumer or caregiver’s positive perception of the credibility or trustworthiness of the information, its professional but friendly tone and pleasant appearance may encourage engagement and systematic processing of the information [1,41].

Evaluation of the quality of clinical guidelines and health information on the internet usually focuses on clinicians’ assessments of the methodological rigor used to develop and report the information [42,43]. However, such assessments, while important, do not tell us anything about the acceptability and usefulness of the information to users [42,43]. Formative evaluations of health information have been conducted to assess and enhance the applicability of this information to users [44,45].

In our study, the panels of clinicians, consumers and caregivers that helped develop the guidelines were selected on the basis that they were very experienced and knowledgeable in dealing with bipolar disorder and the guidelines were compared to the research [29]. However, we did not know how useful the guidelines would be to endusers. Health behavior research suggests that positive appraisal of health information influences positive attitudes and actual use of the information [31,46]. Thus, we invited members of the public who visited the website to give us feedback about the content, design and way the information was conveyed, whether they actually used it, and to suggest improvements.

## Methods

**Figure 1:**
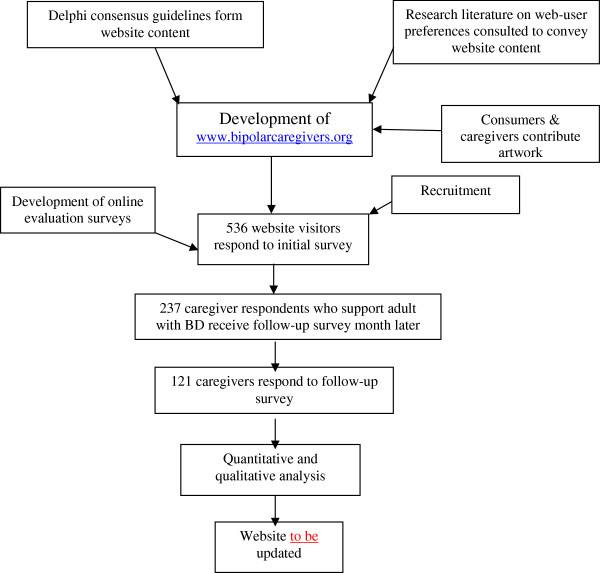
**Development and evaluation of **http://www.bipolarcaregivers.org**.** The figure illustrates the steps taken to develop and evaluate http://www.bipolarcaregivers.org.

The steps from the development of the website for caregivers to its evaluation are illustrated in Figure 1. The website (http://www.bipolarcaregivers.org) is based on a simple WordPress design. To convey the information, we aimed to use non-technical language that was easily understandable. Artwork from consumers with a mental illness and their caregivers helped to illustrate the website. Both web-based and email surveys were used to evaluate the website using the software provider http://www.surveymonkey.com. The ethics application was approved by the ethics committee at the University of Melbourne (ethics ID 0824246).

### Recruitment and participants

The first survey was located on the bipolarcaregivers.org website and visitors who were 18 years or over were invited to respond via a ‘pop-up’ window before they left the site or by going to the feedback page. To participate in the follow-up email survey, participants were required to also be a family member, partner or friend who is a primary support person of an adult with bipolar disorder (18 years or over). To increase the chance of members of the public finding the website and responding to the survey, mental health and caregiver organizations in a number of English speaking developed countries were sent information about the study and, advertisements were placed with permission in public places and in doctors’ waiting rooms over the 13 month recruitment period. In addition, advertisements were placed on Google Ads for a month.

### Measures and procedures

The brief initial survey and more detailed follow-up survey both contained an information sheet outlining the aims and what was involved in participation. Responding to the surveys implied consent.

The aims of the initial survey were to obtain basic information about who was accessing the website and their initial impressions of its usefulness on a four-point scale ranging from ‘Very useful’ to ‘Not useful at all’. Web users were also asked if they would recommend the website to caregivers of adults with bipolar disorder. Participants were encouraged to add comments to clarify their ratings. On completion of the initial survey, expressions of interest were invited from adult caregiver participants of adults with bipolar disorder to give follow-up feedback after a month. The follow-up survey was emailed to these participants a month later and did not require that they revisit the website to respond online.

In the follow-up survey, most questions were structured along a predetermined rating scale, similar to the first survey and participants were encouraged to give comments. Apart from enquiring if caregivers felt more informed, they were asked their appraisals of the usefulness and relevance of the information to themselves and others. Potential barriers to use of the information were assessed by enquiring about how easy the information was to understand and the website to navigate.

To enquire in a preliminary way whether the information affected users’ control attributions and perceptions of social support, we asked whether the information conveyed that: (1) caregivers can help, but the person is essentially responsible for managing their illness; and (2) there is social support out there and that others are dealing with similar challenges.

Besides enquiring about web-user appraisals, we asked about their behavior, specifically whether they had actually used the information (and to give examples). With a dynamic illness like bipolar disorder, participants may not have had the opportunity to use the information over the short follow-up time, and we enquired if they intended to use it in the future. The survey also included open-ended questions about what participants liked most and least about http://www.bipolarcaregivers.org and how it could be improved.

To contextualize the feedback, the follow-up survey examined some caregiver-related and bipolar-disorder-related variables, including the type of caregiver relationship (for example, partner, parent, child, sibling or friend), duration of caregiving experience, whether they lived with the person, as well as questions relating to the chronicity, severity and recency of the person’s bipolar episodes.

A question asking respondents to estimate the amount of time they spent viewing http://www.bipolarcaregivers.org was designed to establish whether they had merely glanced over the site or spent time exploring it. In addition, to maximize response rates, up to three email prompts were sent to participants when necessary [47].

### Quantitative data analysis

Frequency distributions were used to quantify responses to the feedback questions and those assessing caregiver characteristics and situational variables. To address issues related to the generalizability of results, caregivers who responded to the follow-up survey were compared on a few demographic variables, firstly with the broader sample of responders to the initial survey and then to the caregivers of adults with bipolar disorder who only responded to the first survey. The χ^2^ test of independence was used to assess significant differences in categorical variables and an independent sample ttest was used for the continuous variable (age). In addition, where it was necessary to better understand which categories were responsible for significant differences, Wald tests were conducted to examine differences in proportions with Bonferroni adjusted *P* values for multiple comparisons.

### Qualitative data analysis

A content analysis of participants’ comments and suggestions and responses to the open-ended questions provided additional information to clarify and complement the quantitative results. Comments and responses to open-ended questions were categorized into ‘positive appraisals’, ‘negative appraisals’ and ‘ways to improve the website’. These categories were each divided into subcategories of website ‘content’, ‘design’ and ‘way the information was conveyed’. Next, common themes were identified within the subcategories. Some comments were complex and included a number of different themes. For example, one participant’s response to the question about what they liked most about the website was ‘Their openness about the issue and that it is not a death sentence’. This response involved two themes, one about the appraisal of openness and the other about hope. Quantitative and qualitative findings were analyzed in parallel and integrated in the interpretation stage of the study [48].

## Results

Between 3 March 2011 and 12 April 2012, 536 visitors responded to the web-based feedback survey. Of these, 366 (68%) were adult caregivers of adults with bipolar disorder. A total of 237 caregivers agreed to participate in the follow-up survey (65% of those eligible) and 121 finally responded to that survey (51% of those who agreed to participate).

### Participant characteristics

Of the participants, 366 (68.3%) of the respondents to the initial survey were caregivers (primary source of informal support) of an adult with bipolar disorder, 99 (18.5%) were people with bipolar disorder and 98 (18.3%) worked in the area.

Demographic characteristics of users of the website who responded to the initial and follow-up surveys are shown in Table 1. The respondents from both surveys were predominantly female and from Australia. However, those who responded to the follow-up survey were significantly older (although by less than 10 years) than those in the initial survey. They were also more likely to be from Australia and less likely to be from the USA than respondents to the initial survey.

**Table 1:** Gender, Country of origin and age of respondents

**Characteristics**	**Initial survey respondents (N = 536)**	**Follow-up survey (caregivers BD) (N = 121)**
Gender		
Female	428(79.85)	101(83.47)
Location		
Australia^a^	264(49.25)	72(59.50)
USA^a^	178(33.21)	30(24.79)
UK	29(5.41)	6(4.96)
Canada	24(4.48)	5(4.13)
Other^b^	41(7.65)	8(6.61)
Age inyears, mean ± SD^c^	47.36 ± 12.5^d^	53.23 ±11.6

Values are n (%) unless otherwise stated. Caregivers BD are adult caregivers of adults with bipolar disorder.

^a^Statistically significant difference in country of origin.

^b^South Africa, Ireland, France, Italy, Sweden, and Zambia.

^c^Follow-up caregivers BD who were significantly older than the total sample and older than caregivers BD who only responded to the initial survey.

^d^For this category n = 533.

Tables 2 and 3 show that the follow-up sample represented a variety of different types of caregivers dealing with common bipolar disorder-related challenges. Partners and parents of adults with bipolar disorder were more commonly represented (see Table 2). Relatively few caregivers were new to caregiving (9.3% or n = 10). The number of previous bipolar episodes that the person they cared for had experienced varied widely, but only 5 out of the 107 caregivers who responded to this question were dealing with people with bipolar disorder who had experienced their first episode (see Table 3). Most caregivers cared for someone who had experienced a bipolar episode in the last 2 years, but about half cared for someone with an episode in the past month. Nearly 44% of the caregivers reported that the person had experienced four or more bipolar disorder episodes in the past year. Thus, most respondents to the follow-up survey were dealing with recent bipolar episodes and subsyndromal symptoms in those they cared for, and some with a particularly chronic illness course.

**Table 2:** Caregiving characteristics at follow-up

**Caregiving characteristics**	**n**	**%**
Type of caregiver (N = 106):		
Spouse/partner	**43**	**40.6**
Parent	31	29.2
Adult child	17	16
Sibling	7	6.6
Friend	8	7.6
Caregiving duration (N = 107):		
Less than a year	10	9.3
1 to 5 years	29	27.1
6 to 10 years	17	15.9
Over 10 years	**51**	**47.7**
Living arrangements (N = 107):		
Living with carerecipient	**64**	**59.8**

Modal responses are marked in bold.

**Table 3:** Illness characteristics of persons with bipolar disorder (carerecipient) at follow-up

**Care recipient’s bipolar disorder**	**N**	**%**
Number of BD episodes (N = 107)		
1 episode	5	4.7
2 to 5 episodes	26	24.3
6 to 10 episodes	28	26.2
11 or more episodes	**31**	**29.0**
Don’t know	17	15.9
Rapid cycling (N = 107):		
Yes	**47**	**43.9**
Not sure	21	19.6
No	39	36.4
Subsyndromal symptoms (N = 107)		
Yes	**73**	**68.2**
Not sure	19	17.8
No	15	14.0
Recent BD episodes (N = 107)		
Last month:		
Yes	**56**	**52.3**
Not sure	11	10.3
No	40	37.4
Last 2 years:		
Yes	**86**	**80.4**
Not sure	8	7.5
No	13	12.1

Modal responses are marked in bold.

### Quantitative feedback

#### Usefulness of the content

A total of 97% (n = 520) of web users who responded to the initial survey (N = 536) reported that they thought the information was ‘very useful’ (56.7% or n = 304) or ‘useful’ (40.3% or n = 216) to adult caregivers of adults with bipolar disorder. Under 3% (n = 15) thought it was ‘not very useful’ and one person found it ‘not useful at all’. Table 4 shows that high endorsement of the usefulness of the information was also reported by follow-up respondents (N = 121). The usefulness of each of the website sections was viewed in the context of what participants read. When looking at ‘total’ useful ratings (very useful + useful), the sections on ‘Bipolar Disorder’, ‘Providing Support’ and ‘Treatment/Management’ were rated as useful by around 95% or more of the caregivers who read those sections. These three sections were also the most popular. All sections were rated as useful by over 85% of those who read them.

**Table 4:** Caregivers’ ratings of the usefulness of the information they read at follow-up

**Website section/PDF (N = 121)**^**a**^	**Very useful, % (n)**	**Useful, % (n)**	**Total of very useful + useful, % (n)**	**Total of not that useful + not useful at all, % (n)**
Bipolar disorder, n = 116,	48.3 (56)	**49.1 **(57)	97.4 (113)	2.6 (3)
Providing support, n = 117	**52.1 (61)**	43.6 (51)	95.7 (112)	4.3 (5)
Treatment/management, n = 113	41.6 (47)	**54.0 (61)**	95.5 (108)	4.4 (5)
Information summaries, n = 96	38.5 (37)	**55.2 (53)**	93.7 (90)	6.3 (6)
Caregiver self-care, n = 110	44.5 (49)	**49.1 (54)**	93.6 (103)	6.4 (7)
PDF guide for caregivers, n = 110	**46.4 (51)**	**46.4 (51)**	92.8 (102)	7.3 (8)
Working together, n = 110	39.1 (43)	**52.7 (58)**	91.8 (101)	8.2 (9)
Resources, n = 109	35.8 (39)	**55.0 (60)**	90.8 (99)	9.1 (10)
Stigma/discrimination, n = 103	37.9 (39)	**48.5 (50)**	86.4 (89)	13.6 (14)

Table 4 shows the percentage of caregivers who found each of the sections of the website they read very/ useful or not that useful/not useful at all. Modal responses are marked in bold.

^a^n = number of caregivers who read section/PDF.

High numbers of respondents to the follow-up survey reported having read the website sections (see Table 4). In addition, the most commonly reported timeframe for viewing the website was an hour, with a mean of 1.39 h and SD 2.8 h (n = 94) and variable range (10 minutes to 20 h). Taken together with the amount of qualitative feedback provided, these results suggest that caregivers who participated in the follow-up survey had reviewed the content to some extent.

### Relevance to participants

In response to the follow-up survey, 92.6% (n = 113) of the 121 caregivers reported that the information was definitely (62% or n = 75) or moderately (30.6% n = 37) relevant to their situation. The rest found it slightly relevant (6.6% or n = 8) or not relevant at all (n = 1).

### Usefulness and relevance to others

In response to the initial survey, about 89.7% (n = 481) of the 536 respondents said they would recommend the website to caregivers of people with bipolar disorder, 9% (n = 48) said they were not sure and 1.3% (n = 7) said they would not recommend it. An even higher number of caregivers at follow-up (95.9% n = 116) reported that they thought other caregivers might find the information helpful.

### Content was informative

About 62% (n = 75) of the 121 caregivers at follow-up thought that the information helped them to be more informed and knowledgeable and 34.7% or n = 42 said ‘to some extent’.

### Content was supportive

All caregivers at follow-up (N = 121) reported that the information confirmed that other caregivers were going through similar experiences to them (90.9% or n = 110 said ‘yes’ and 9.1% or n = 11 said ‘to some extent’). A total of 99% said it gave them, at least to some extent, the impression that there was support for caregivers out there (72.7% or n = 88 said ‘yes’ and 26.4% or n = 32 said ‘to some extent’).

### Helped with caregiver control appraisals

Of the 121 caregivers at follow-up, 99% reported that the information gave them the impression that there are ways caregivers can help, although the person with bipolar disorder is ultimately responsible for managing their own illness (79.3% or n = 96 said ‘yes’ and 19.8% or n = 24 said ‘to some extent’).

### Easy to understand

Almost 56% or 60 respondents to this question on the follow-up survey (N = 108) reported that the information on the website was ‘very easy’ to understand, and 43.5% or 47 that it was ‘easy’ to understand.

### Easy to navigate

Half of the 108 respondents to this question on the follow-up survey reported that the website was 'very easy' to navigate and 49.1% said that it was ‘easy’.

### Actual use of information

Table 5 shows reports from caregivers at follow-up (N = 108) about their actual use of the various content areas they read. Nearly three-quarters (72% or n = 77) reported using the section on providing support and over 60% the sections on working together and caregiver self-care.

**Table 5:** Percentage of caregivers who reported using the information they read

**Website content areas (N = 108)**^**a**^	**Yes, % (n)**	**Not sure, % (n)**	**No, % (n)**
Providing support, n = 107	**72.0 (77)**	17.8 (19)	10.2 (11)
Working together, n = 106	**67.9 (72)**	15.1 (16)	17.0 (18)
Self-care, n = 104	**63.5 (66)**	17.3 (18)	19.2 (20)
Stigma/discrimination, n = 99	**46.5 (46)**	20.2 (20)	33.3 (33)
Resources, n = 98	40.8 (40)	16.3 (16)	**42.9 (42)**

This table shows the percentage of caregivers who reported using, not using or being unsure whether they used the information on the website. The first column shows the section of the website and number of caregivers who read that section. Modal responses are marked in bold.

^a^n = respondents who read the information.

### Intentions to use information

In all, 92 participants in the follow-up survey (N = 108) or 85.2% said they would use the information in the future. A total of 13 respondents or 12% said that they were not sure if they would use the information. Three respondents or 2.8% said they would not use the information in the future.

### Qualitative feedback

There were 316 comments made by participants in response to the first survey and over 250 received in response to the follow-up survey. As there was a lot of repetition of qualitative responses between the initial and follow-up surveys, common themes are reported below and the few differences highlighted. Numerous general comments were made and have been excluded from the analysis (for example, ‘an excellent resource!’). (Words in quotation marks are the actual words of respondents.)

### Positive appraisals

Examples of specific positive themes and comments include (number in brackets refers to incidences theme mentioned):

• Particular content appreciated (n = 37): (for example, bipolar disorder symptoms, treatments, ways to support and communicate with person, dealing with treatment refusal, caregiver self-care, common caregiver emotions and establishing healthy boundaries, resources, printable guidelines and summaries).

• Helped with sense of control (n = 2): (for example, ‘I found it incredibly supportive, empowering and useful in my own situation’).

• Supportive, empathic and validating (n = 36): (for example, ‘Helps that you do not feel alone’, ‘Gives a feeling of ‘family’-empathy and hope’, ‘It is ok to have feelings of frustration’, ‘I knew a lot of this, but it is helpful to get confirmation’ and ‘it was reassuring’).

• Appreciated openness about commonly stigmatized topics (n = 2): (for example, ‘has the answers to the questions I was too afraid to ask’).

• Conveyed a hopeful perspective (n = 2). (for example, ‘not a death sentence’ and ‘information boosted my optimism’).

• Good quality content (n = 30) (for example, information is ‘trustworthy’, ‘credible’, ‘practical’, ‘reliable’, ‘non-biased’ and ‘broad range of information’).

• Addressed need for information: (n = 17) (for example, liked ‘bipolar disorder-specific information’ and ‘Information given specifically for caregivers, from our point of view’).

Positive appraisals of the design of the website and way the information was conveyed from responses to both surveys included:

• Easy to understand (n = 34) (for example, ‘clear’, ‘easy to read and understand’).

• Easy to navigate (n = 15) (for example, ‘easy to find what I was looking for’ and ‘broken into small ‘chunks’ of information, which can be easily accessed if someone is stressed or has little time’).

• Appreciated tone (n = 11) (for example, ‘friendly’, ‘not too confronting’, ‘non-judgmental’, ‘not patronizing’ and ‘wonderful professionalism and directness as needed’).

• Appreciated presentation (n = 7) (for example, ‘Very clear fonts and colors, no adverts’ and ‘loved the artwork’).

• Online information convenient (n = 20) (for example, ‘easy to access’ and ‘availability to read in your own space’).

### Negative appraisals

Examples of specific negative appraisals given in response to initial and follow-up surveys included:

• Content too general or basic (n = 22) This was usually reported by web users who already very knowledgeable and experienced or who are caring for person with very severe chronic bipolar disorder or family problems (for example, ‘would have made a huge difference to me when we were first dealing with a family member who developed bipolar disorder’ and ‘The website is way too generic …. I care for an ultra-rapid cycler. I really need a shoulder to cry on’ and ‘how can one reflect, forgive, start over, when the loved one is resisting contact?’). Alternatively, one participant mentioned that the person’s bipolar disorder was ‘very much currently under control’ so they found the website less relevant.

• Need for specific information (n = 15) (for example, specific information on medications without sexual or sleep-related side effects, local housing and occupation options, helping children of bipolar parents and dealing with family problems that are independent of ‘a bipolar crisis’).

Negative appraisals of design and how the information was conveyed included:

• Navigation difficulties (n = 5) (for example, ‘too complex’ and ‘I felt like I was being attacked by the pop-up’).

• Difficult to understand (n = 3) (for example, ‘literacy level might be too high for others’)

• Presentation problems (n = 3) (for example, ‘too sanitized’ or ‘too many fonts and colors on pages’).

In addition, four respondents to the initial survey found the information too based on the medical model (for example, ‘Extremely inaccurate and medical model based’). Another suggested that caregivers should always take an active stand against stigma whereas the Delphi panels recommended that caregiver assess what to do in the particular situation. Some respondents wanted specific information for child/adolescent caregivers, childhood bipolar disorder or caring for people with other mental health conditions.

### Suggestions for improvement

There were also comments about providing additional or more in-depth information on topics such as ‘bipolar II disorder’, ‘the science behind bipolar disorder’, ‘reducing stress’ and links to more resources. A total of 12 people recommended enhancing the supportive aspect of the website by making it more interactive (for example, ‘support forum’ or ‘personalized support aspect’), having templates for plans and coping skills, anonymous personal stories and examples of how to apply suggestions. Four respondents mentioned that they thought the content should be more widely disseminated by advertising the website URL in more public places or creating a booklet and leaflets for caregivers based on the guidelines. The authors have also had many requests from web users via email for a booklet version of the guidelines.

### Use of website content

Examples of how participants used the sections of the website included:

• Providing support (n = 2) (for example, ‘helping someone with depression and recognizing risk factors’).

• Working together to manage the illness (n = 9): Examples include communicating differently when the person was ill (for example, ‘deferring topics of conversation’) and discussing illness management (for example, when person wants to ‘stop medications’, dealing with ‘manic triggers’ and ‘ways to help the person without mentioning the illness’).

• Caregiver self-care (n = 10): There were comments about recognizing the need and implementing self-care (for example, ‘Finally realized I need to take care of myself’, ‘Have begun to manage my own ‘time out’ & relieving my own stress issues by learning to remove myself from the situation at a more appropriate time’, ‘Reached out for help for me, worked to maintain my contacts better’). A number of respondents mentioned learning to set boundaries (for example, ‘have been given the strength to just step back a little … and …the courage for not taking responsibility where I shouldn’t - without feeling the guilt by doing so.’

• Dealing with stigma and disclosure (n = 1): (for example, ‘My previous attitude of not telling people because we had a horrible shameful secret was changed to being simply a practical matter of being selective because of how people may wrongly change their attitude towards the person with bipolar disorder’).

• Resources (n = 4): A number of people reported accessing books and one accessed a society.

• Potential impact on relationships (n = 3) (for example, ‘The impact this website has had on how to take care of myself has really boosted my optimism in seeing that it is possible to maintain the relationship….’).

Some respondents said the information confirmed the way they were already coping and others mentioned not having the opportunity to use the information in the short follow-up time.

## Discussion

Overall, the results suggest that most respondents found the information relevant, useful and considered it would be helpful to other caregivers. As Jorm [49] has noted, ‘mental health literacy is not simply a matter of having knowledge (as might be conveyed in an abnormal psychology course). Rather it is knowledge that is linked to the possibility of action to benefit one’s own mental health or that of others’. Given the relatively short follow-up period and erratic course of bipolar disorder, a surprising number of caregivers reported using the various sections of the website and gave concrete examples of this use.

It was not surprising that most respondents reported that the information was relevant to their situation as they were dealing with relatively recent and dynamic symptoms typical of bipolar disorder. According to the Transtheoretical Model of change conceptualized by Prochaska and DiClemente [50], when people are in the precontemplation stage they are not aware of the importance of changing a behavior, but awareness grows as they proceed to the contemplation stage and weigh up the costs and benefits of changing. In the preparation stage the person makes plans to change and this is succeeded by the action and maintenance stages. The targeted content based on the Delphi study may have increased active contemplation of the information on the website.

Other studies have highlighted the value of including consumer, caregiver and clinician stakeholders in the development and evaluation of health information [51,52]. The method used to develop the website content in our study was similar to the Delphi consensus method involving expert stakeholders used to develop Mental Health First Aid (MHFA) guidelines with information for the public on recognizing and responding to mental health problems and crises up to the point that professional help is accessed [49]. Hart and colleagues [51] also used online surveys to evaluate the usefulness of the MHFA guidelines. Around 80.1% of the web users involved in the follow-up evaluation of the online MHFA guidelines rated the guidelines they downloaded as either very useful or useful, and 83.8% said they were very likely or likely to use them in the future [51]. Our study supports this and other research [44,45,53,54] that highlight the value of engaging users in the development and evaluation of health information.

Web user feedback about http://www.bipolarcaregivers.org emphasized the usefulness of information about bipolar disorder, treatment and management and ways the caregiver can communicate with the person that have been highlighted in both psychosocial interventions and studies of expressed emotion [55]. In addition, a number of web users commented on the value of information that addressed ways to manage particular real life challenges when providing support, such as what caregivers can do if the person refuses to manage or treat their bipolar disorder.

Nearly all respondents reported the information conveyed that there were things caregivers can do to help, although the person is responsible for their illness. Caregivers may need to be more actively involved in caregiving at times, but trying to control the person’s illness may impact negatively on their relationship with the person, caregiver burden and the course of bipolar disorder [14,21,56]. The idea is to address the person’s basic psychological needs for autonomy, competence and relatedness within the caregiving circumstances as much as possible (for example, making advance plans with the person about what to do in a bipolar crisis) [57]. Knowing how to help in the different circumstances may assist caregivers to feel a greater sense of control. However, our results suggest that their sense of control over their situation may also be influenced by knowing how to set realistic limits with the person and their caregiving role (for example, ‘It helped me to know that I do not have to take her verbal abuse and how to deal with’). One of the ways to reduce caregiver resentment and anger is not simply for caregivers to be informed and accept that a person’s problematic behavior is due to their illness, but to have their own needs acknowledged and be able to set some limits on what behavior they will tolerate [58].

Possibly, some caregivers saw the information on caregiver self-care as giving them permission to attend to their own basic psychological and health related needs as well as the person’s. Although health outcomes were not measured, more positive attitudes towards self-care and increases in positive health behavior may enhance caregiver health and wellbeing [59]. In addition, recognizing the importance of their own needs as well as the person’s may have a positive influence on the caregiver’s relationship with the person with bipolar disorder (for example, ‘The impact this website has had on how to take care of myself has really boosted my optimism about continuing’).

Although a considerable percentage of respondents said the information on the website helped them feel better informed, it may have had other functions. All follow-up participants reported that the information reassured them about the universality of their emotions and experience and some commented that it helped them to feel less alone and isolated. Information that highlights the universality of common emotions may help caregivers to make sense of their reactions (for example,‘realized that it is ok to have feelings of frustration, and that it doesn’t mean I don’t love the person’). Isolation may contribute to caregiver depression [60]. A high percentage of respondents reported that it gave them the impression that there is support out there. Although perceptions of support were not formally measured, feedback from respondents suggests that some web users found it emotionally supportive (for example, ‘non-judgmental’, ‘empathic’, ‘reassuring’ and ‘supportive’ and ‘they understand and care’. The social support literature suggests that perceptions of support can help to regulate emotions and the negative effects of stressful situations [61-63].

The perception of empathy may also be enhanced by “information seeking effectiveness”, the extent to which online information is perceived as ‘readily usable’, ‘credible’, ‘relevant’, ‘reliable’ and accessible in a ‘timely manner’ [64]. A recent study of a forum where people with cancer and caregivers posted and responded to healthcare queries found that ‘information seeking effectiveness’ was more predictive of perceptions of empathy by participants than the social support aspects (for example, being able to talk about problems) [64]. Perceptions of homophily (perceptions of others as similar or as sharing similar life experiences) moderated the connection between information-seeking effectiveness and perceived empathy. Most web users in our study also reported finding the website content easy to access, practical, relevant, credible, reliable and they appreciated the tone and appearance of the website. Communication that is perceived as empathic may help caregivers to feel supported and encourage them to actively contemplate the information and weigh up the benefits of using it [65-67].

While overall the evaluation of http://www.bipolarcaregivers.org was very positive, participants pointed out what they did not like about the website and how it could be improved, suggesting that they were motivated to give accurate rather than socially desirable feedback. For example, some caregivers who were already knowledgeable and experienced found the content less relevant to their situation. Possibly, the website may have offered slightly different benefits to different caregivers. Although the website may have provided new information to more recent caregivers, others reported the information was ‘reassuring’ and ‘confirmed’, ‘validated’ and ‘reinforced’ what they already knew. Nevertheless, comments from a few experienced or knowledge web users indicate that they may be looking for more specific information, such as what medication does not have a certain side effect, or more detailed information, such as updates about scientific research on specific aspects of bipolar disorder.

Qualitative feedback from some of the caregivers who were dealing with very severe and chronic forms of bipolar disorder, showed that the website did not fully satisfy their need for information or emotional support (for example, ‘My husband is an ultra-rapid cycler and does not respond well to many of the drugs currently available. It is difficult for us both and I really need a shoulder to cry on’). They wanted specific local information on social resources and health services. Challenges faced by caregivers of people with multiple or frequent episodes, comorbidity and poor interepisode recovery, as defined in staging models [68,69], may be very different from those caring for a person with much less severe illness [70]. In addition, families dealing with conflict, breakdown and crises in relationships, may need more active help than can be provided on a passive information website [71]. Ideally, health services could have a role in providing individually tailored information and support to cater for the variety of caregiver needs over the illness trajectory, but this is not usually possible in practice [2]. Perhaps, http://www.bipolarcaregivers.org could form part of a stepped-care approach to supporting caregivers tailored to their needs, preferences and the accessibility of more specialized and individualized psychosocial interventions for families and caregivers [25].

A more interactive version of the website with options for caregivers to tailor information and support to their individual needs was recommended by some respondents. Similarly, in a focus group study of user feedback about websites for physical illness, patients and caregivers appreciated being able to select what information was relevant to them, as on our website, but valued having some online interactivity such as personalized assessments with advice from experts or peer support [1]. Such interactivity comes at a financial cost, but could be a way to help individualize information and provide additional support on http://www.bipolarcaregivers.org in the future.

Currently, http://www.bipolarcaregivers.org may reduce some of the barriers the public have to accessing mental health information and enhance mental health literacy [25,49,70]. Perceived stigma has been reported to sometimes obstruct information seeking about mental health problems [72,73]. In our study, participants commented on the ‘openness’ of the information in answering questions they were ‘too afraid to ask’. Our research confirms the convenience for caregivers of accessing online health information when it suits them in the privacy of their home for busy caregivers in the privacy of their own home [74,75]. Another advantage is that participants can return for new or ‘booster’ information at any time along the illness trajectory [2]. At the time of writing, the site is receiving around 5,000 visits a month according to Google Analytics, and nearly a fifth of these are from returning visitors. On a public health level, this resource may fill an important gap in providing basic information and reassurance for caregivers that influences positive actions.

The results need to be interpreted in the context of study limitations. In the future, more formal assessment of caregiver factors, such as burden, knowledge, mastery and perceived support, as well as prospective health outcomes, would provide a more rigorous evaluation. Comparing http://www.bipolarcaregivers.org to a leaflet, or alternatively to a more interactive website over a longer follow-up time could confirm the internal validity of results. In addition, in the current study it was not logistically possible to conduct the evaluation by an independent team rather than the team who developed the website, but in future this would strengthen the findings.

Another limitation of the current evaluation study was that the number of web users who responded to our surveys was relatively small, given that over the recruitment period 20,379 unique visitors accessed the website (according to Google Analytics). Low response rates are common with online surveys [76]. While we cannot be certain that our sample represents this larger visitor population, it did include a variety of caregivers of people with bipolar disorder, consumers and those who work in the area. Selection bias may also have been introduced in the follow-up sample, as these comprised only 33% of the eligible caregiver respondents from the initial survey, and might have reflected the views of those who were more favorably disposed to the website (therefore more likely to complete the follow-up survey). Furthermore, there were significant differences in the age and location of web users who responded to the initial and follow-up surveys. While most respondents in both the initial and follow-up surveys rated the information positively, the follow-up study also found that caregivers contributed negative appraisals and suggestions for improvement, suggesting that they did not only view the website positively. These caregivers were dealing with a range of challenges highlighted in the literature regarding caregivers of people with bipolar disorder, and the richness of their responses suggests a genuine need for information and support to deal with these challenges rather than only a positive bias. Thus, while caution should be exercised regarding the generalizability of the results, this evaluation study offers preliminary evidence that the website content was overall both appraised positively and actually used by a number of adult caregivers of adults with bipolar disorder dealing with common caregiving challenges. It also indicated concrete ways to improve this resource.

More specific or detailed information (for example, on bipolar II disorder and the biological causes of bipolar disorder, ways to reduce stress) will be added to http://www.bipolarcaregivers.org together with templates, examples and links to resources in accordance with the feedback. Although desirable [37], more interactive ways to tailor information to users’ needs and provide support will depend on future funding. Such funding might also facilitate the development of similar resources for other caregiver groups (for example, caregivers of people with different disorders, younger caregivers, those from different cultures or who have minimal access to functional health services).

## Conclusions

A strength of this evaluation was its mixed method design that facilitated insight into the overall usefulness of the information, as well as specific improvements that could be made. A high percentage of web users who gave feedback appraised the information positively and some actually used it over the follow-up month. Besides being informative, the website may have helped caregivers to perceive a greater sense of control over their situation, to set boundaries with the person and their caregiving role, and to feel supported and validated. However, some caregivers who already knew the information, who were very experienced or were dealing with severe bipolar disorder or problematic family situations did not find the information as useful. There is no ‘one-size-fits-all’ solution to supporting caregivers of people with bipolar disorder and a stepped care approach that facilitates needed access to more specialized interventions is required. However, despite limitations, this evaluation offers preliminary evidence of the usefulness of http://www.bipolarcaregivers.org as a publically accessible resource for adult caregivers in need of general but practical information, links to resources and reassurance. Information that supports caregivers in carrying out their vital informal role and maintaining their own wellbeing may positively impact on the person with bipolar disorder and their relationship.

## Abbreviations

ARHRF: Australian Rotary Health Research Fund; MHFA: Mental Health First Aid; NHMRC: National Health and Medical Research Council.

## Competing interests

SD has received grant/research support from the Stanley Medical Research Institute, National Health and Medical Research Council (NHMRC), Beyond Blue, Australian Rotary Health Research Fund (ARHRF), Simons Foundation, Geelong Medical Research Foundation, Eli Lilly, Glaxo SmithKline, Organon, Mayne Pharma and Servier, speaker’s and advisory board fees from Eli Lilly and conference travel support from Servier. MB has received grant/research support from the Stanley Medical Research Institute, Medical Benefits Fund, NHMRC, Beyond Blue, Geelong Medical Research Foundation, ARHRF, Bristol Myers Squibb, Eli Lilly, Glaxo SmithKline, Organon, Novartis, Mayne Pharma, Servier and Astra Zeneca, consultant fees from Astra Zeneca, Bristol Myers Squibb, Eli Lilly, Glaxo SmithKline, Janssen Cilag, Lundbeck and Pfizer and speaker’s fees from Astra Zeneca, Bristol Myers Squibb, Eli Lilly, Glaxo SmithKline, Janssen Cilag, Lundbeck, Organon, Pfizer, Sanofi Synthelabo, Solvay and Wyeth. The other authors have no competing interests.

## Authors’ contributions

LB developed and evaluated the website as part of her PhD project, drafted the manuscript and coordinated edits from other authors. MB acted as a consultant on the project. SD and CMK helped supervise the project and SC contributed to the data analysis. AFJ provided regular supervision and input in the design and implementation of the project. All authors were involved in editing the manuscript, and read and approved the final version.

## Pre-publication history

The pre-publication history for this paper can be accessed here:

http://www.biomedcentral.com/1741-7015/11/162/prepub
